# Comparison of long-read sequencing technologies in the hybrid assembly of complex bacterial genomes

**DOI:** 10.1099/mgen.0.000294

**Published:** 2019-09-04

**Authors:** Nicola De Maio, Liam P. Shaw, Alasdair Hubbard, Sophie George, Nicholas D. Sanderson, Jeremy Swann, Ryan Wick, Manal AbuOun, Emma Stubberfield, Sarah J. Hoosdally, Derrick W. Crook, Timothy E. A. Peto, Anna E. Sheppard, Mark J. Bailey, Daniel S. Read, Muna F. Anjum, A. Sarah Walker, Nicole Stoesser, on behalf of the REHAB consortium, M Abuoun, M Anjum, M Bailey, H Brett, M Bowes, K Chau, DW Crook, N de Maio, N Duggett, D Gilson, HS Gweon, A Hubbard, S Hoosdally, J Kavanaugh, H Jones, TEA Peto, DS Read, R Sebra, LP Shaw, AE Sheppard, R Smith, E Stubberfield, N Stoesser, J Swann, AS Walker, N Woodford

**Affiliations:** ^1^​ Nuffield Department of Medicine, University of Oxford, Oxford, UK; ^2^​ Department of Tropical Disease Biology, Liverpool School of Tropical Medicine, Liverpool, L3 5QA, UK; ^3^​ NIHR HPRU Health Protection Research Unit in Healthcare Associated Infections and Antimicrobial Resistance at University of Oxford in partnership with Public Health England, Oxford, UK; ^4^​ Department of Biochemistry and Molecular Biology, Bio21 Molecular Science and Biotechnology Institute, University of Melbourne, Melbourne, Australia; ^5^​ Department of Bacteriology, Animal and Plant Health Agency, Addlestone, Surrey, KT15 3NB, UK; ^6^​ Centre for Ecology & Hydrology, Benson Lane, Crowmarsh Gifford, Wallingford, OX10 8BB, UK

**Keywords:** hybrid assembly, bacterial genomics, long-read sequencing, *Enterobacteriaceae*, plasmid assembly

## Abstract

Illumina sequencing allows rapid, cheap and accurate whole genome bacterial analyses, but short reads (<300 bp) do not usually enable complete genome assembly. Long-read sequencing greatly assists with resolving complex bacterial genomes, particularly when combined with short-read Illumina data (hybrid assembly). However, it is not clear how different long-read sequencing methods affect hybrid assembly accuracy. Relative automation of the assembly process is also crucial to facilitating high-throughput complete bacterial genome reconstruction, avoiding multiple bespoke filtering and data manipulation steps. In this study, we compared hybrid assemblies for 20 bacterial isolates, including two reference strains, using Illumina sequencing and long reads from either Oxford Nanopore Technologies (ONT) or SMRT Pacific Biosciences (PacBio) sequencing platforms. We chose isolates from the family *Enterobacteriaceae*, as these frequently have highly plastic, repetitive genetic structures, and complete genome reconstruction for these species is relevant for a precise understanding of the epidemiology of antimicrobial resistance. We *de novo* assembled genomes using the hybrid assembler Unicycler and compared different read processing strategies, as well as comparing to long-read-only assembly with Flye followed by short-read polishing with Pilon. Hybrid assembly with either PacBio or ONT reads facilitated high-quality genome reconstruction, and was superior to the long-read assembly and polishing approach evaluated with respect to accuracy and completeness. Combining ONT and Illumina reads fully resolved most genomes without additional manual steps, and at a lower consumables cost per isolate in our setting. Automated hybrid assembly is a powerful tool for complete and accurate bacterial genome assembly.

## Data Summary

Raw sequencing data have been deposited under NCBI BioProject Accession PRJNA422511 (https://www.ncbi.nlm.nih.gov/bioproject/PRJNA422511). Assemblies and supplementary figures and tables are available on FigShare (https://figshare.com/articles/Hybrid_Enterobacteriaceae_assemblies_using_PacBio_Illumina_or_ONT_Illumina_sequencing/7649051). We confirm all supporting data, code and protocols have been provided within the article or through supplementary data files.

**Impact Statement**

Illumina short-read sequencing is frequently used for tasks in bacterial genomics, such as assessing which species are present within samples, checking if specific genes of interest are present within individual isolates, and reconstructing the evolutionary relationships between strains. However, while short-read sequencing can reveal significant detail about the genomic *content* of bacterial isolates, it is often insufficient for assessing genomic *structure*: how different genes are arranged within genomes, and particularly which genes are on plasmids – potentially highly mobile components of the genome frequently carrying antimicrobial resistance elements. This is because Illumina short reads are typically too short to span repetitive structures in the genome, making it impossible to accurately reconstruct these repetitive regions. One solution is to complement Illumina short reads with long reads generated with SMRT Pacific Biosciences (PacBio) or Oxford Nanopore Technologies (ONT) sequencing platforms. Using this approach, called ‘hybrid assembly’, we show that we can automatically fully reconstruct complex bacterial genomes of *Enterobacteriaceae* isolates in the majority of cases (best-performing method: 17/20 isolates). In particular, by comparing different methods we find that using the assembler Unicycler with Illumina and ONT reads represents a low-cost, high-quality approach for reconstructing bacterial genomes using publicly available software.

## Introduction

The rapid development of microbial genome sequencing methods over the last decade has revolutionized infectious disease epidemiology, and whole genome sequencing has become the standard for many molecular typing applications in research and public health [1–4]. Much of this evolution has been driven by the development of high-throughput, low-cost, second-generation (short-read) sequencing methods, such as Illumina’s HiSeq and MiSeq platforms, which produce millions of low-error (0.1%) paired-end reads, generally 100–300 bp in length. As such, Illumina sequencing has become the most widely used sequencing technology for microbial genomics. Multiple read processing algorithms now exist, typically enabling variant detection following mapping to a reference genome to assess genetic relatedness (e.g. for outbreak investigation or population genetic studies), or *de novo* assembly to facilitate the identification of important loci in the accessory genome, such as antimicrobial resistance (AMR) genes (e.g. for epidemiological studies of resistance gene prevalence or for susceptibility prediction).

However, it has become clear that short-read sequencing has significant limitations depending on the bacterial species and/or epidemiological question. These limitations arise largely from the inability to fully reconstruct genomic structures of interest from short reads, including both those on chromosomes and on mobile genetic elements such as plasmids [5]. An example where this genomic structure is highly relevant is the study of AMR gene transmission and evolution in species of *Enterobacteriaceae *, which have emerged as a major clinical problem in the last decade [6]. Short-read data from these species do not successfully facilitate assembly of the repetitive structures that extend beyond the maximum read length generated, including structures such as resistance gene cassettes, insertion sequences and transposons that are of crucial biological relevance to understanding the dissemination of key AMR genes.

The most widely used single-molecule, long-read sequencing platforms, currently represented by Pacific Biosciences' (PacBio) Single Molecule Real-Time (SMRT) and Oxford Nanopore Technologies' (ONT) MinION sequencers, are often able to overcome these limitations by generating reads with a median length of 8–10 kb and as long as 100 kb [5, 7, 8]. However, the sequencing error rates of both long-read platforms have typically been greater than for Illumina platforms, although they have decreased over recent years and continue to do so. As a guide, PacBio errors have been estimated at 11–15 % for the single pass method [9] and significantly less in circular consensus reads, which can achieve ~0.001 % error leading to assemblies at >Q50 [10]; for ONT, a recent meta-analysis put the error range between 5 and 15 %, varying by chemistry and base caller used [11]. Hybrid assembly, using combined short-read and long-read sequencing datasets, has emerged as a promising approach to generating fully resolved and accurate bacterial genome assemblies. With hybrid approaches, long reads provide information regarding the structure of the genome and short reads facilitate detailed assembly at local scales, and can be used to correct errors in long reads [12–14]. The hybrid assembly tool Unicycler has been shown to outperform other hybrid assemblers in generating fully closed genomes [13].

We are not aware of any previously published direct comparisons of hybrid bacterial assemblies generated using long-read sequencing methods, yet the selection of a long-read sequencing approach has important cost, throughput and logistical implications. Currently, the two dominant long-read technologies are ONT and PacBio. The ONT MinION is a highly portable platform that has been deployed in many molecular laboratories, including those in low-income settings [15]. Reported data yields of 10–30 Gb and indexed barcoding have enabled successful assembly with multiplexing of 12 bacterial isolates [14, 16]: a 24 barcode ligation kit has recently been released by ONT, and a recent report outlined successful assembly with 48 clinical *Staphylococcus aureus* isolates per flow cell without barcoding [17], although this may not be effective for closely related isolates with plasmids. In contrast, the PacBio platform is non-portable but has been the most widely used for generating reference-grade bacterial assemblies to date, such as in the NCTC 3000 Project [18] (by way of example: as of 21 January 2019, the NCBI Assembly database contained 201 *Escherichia coli* assemblies generated with PacBio vs. three generated with MinION).

Here we compared different approaches for hybrid bacterial genome assembly, using ONT MinION, PacBio and Illumina HiSeq data generated from the same DNA extracts. We selected 20 bacterial isolates from four genera of the family *Enterobacteriaceae* (* Escherichia *, *Klebsiella *, *Citrobacter* and *Enterobacter *) including two reference strains. These genera typically have large bacterial genomes of 4–6.5 Mb with diverse sets of plasmids [19]. We compared the advantages and disadvantages of ONT+Illumina versus PacBio+Illumina hybrid assembly, including the need for additional manual processing steps, and compared these assemblies with those generated by using long-read assembly (Flye) followed by polishing with Illumina data (Pilon). We also investigated different strategies to optimize hybrid assembly using Unicycler for both long-read approaches.

## Methods

### Bacterial isolates, DNA extraction and Illumina sequencing

For sequencing, we selected and subcultured 20 isolates across the four genera of interest from stocks of pure culture, stored in nutrient broth with 10 % glycerol at −80 °C. Subcultures were undertaken aerobically on Columbia blood agar at 37 °C overnight. We chose two reference strains, *Escherichia coli* CFT073 (NC_004431.1) and *Klebsiella pneumoniae* MGH78578 (NC_009648.1-NC_009653.1), and 18 isolates that were part of a study investigating antimicrobial resistance in diverse *Enterobacteriaceae* from farm animals and environmental specimens (the REHAB study, http://modmedmicro.nsms.ox.ac.uk/rehab; details of isolates in Table S1). These comprised *E. coli* (*n*=4), *K. pneumoniae* (*n*=2), *K. oxytoca* (*n*=2), *Citrobacter freundii* (*n*=2), *C. braakii* (*n*=2), *C. gillenii* (*n*=1), *Enterobacter cloacae* (*n*=3) and *E. kobei* (*n*=2). We chose to investigate *Enterobacteriaceae* isolates as these bacteria are genetically complex: their genomes commonly contain multiple plasmids and repeat structures of varying size, making them difficult to assemble using other methods [5].

DNA was extracted from subcultured isolates using the Qiagen Genomic tip 100/G kit (Qiagen) to facilitate long-fragment extraction. Quality and fragment length distributions were assessed using the Qubit fluorometer (ThermoFisher Scientific) and TapeStation (Agilent).

All DNA extracts were sequenced using the Illumina HiSeq 4000, generating 150 bp paired-end reads. Libraries were constructed using the NEBNext Ultra DNA Sample Prep Master Mix Kit (NEB) with minor modifications and a custom automated protocol on a Biomek FX (Beckman Coulter). Ligation of adapters was performed using Illumina Multiplex Adapters, and ligated libraries were size-selected using Agencourt Ampure magnetic beads (Beckman Coulter). Each library was PCR-enriched with custom primers (index primer plus dual index PCR primer [20]). Enrichment and adapter extension of each preparation was obtained using 9 µl of a size-selected library in a 50 µl PCR. Reactions were then purified with Agencourt Ampure XP beads (Beckman Coulter) on a Biomek NXp after 10 cycles of amplification (as per Illumina recommendations). Final size distributions of libraries were determined using a TapeStation system as above and quantified by Qubit fluorometry.

### ONT library preparation and sequencing

ONT sequencing libraries were prepared by multiplexing DNA extracts from four isolates per flowcell using the SQK-LSK108 and EXP-NBD103 kits according to the manufacturer's protocol with the following amendments: input DNA (1.5 µg) was not fragmented, 2 ml Eppendorf DNA LoBind tubes (Eppendorf) were used, all reactions were purified using 0.4× Agencourt AMPure XP beads, incubation time with Agencourt AMPure XP beads was doubled, elution volumes were reduced to the minimum required for the subsequent step, and elution was heated to 37 °C. Libraries were loaded onto flow cell versions FLO-MIN106 R9.4 SpotON and sequenced for 48 h.

### PacBio library preparation and sequencing

DNA extracts were initially sheared to an average length of 15 kb using g-tubes, as specified by the manufacturer (Covaris). Sheared DNA was used in SMRTbell library preparation, as recommended by the manufacturer. The quantity and quality of the SMRTbell libraries were evaluated using the High Sensitivity dsDNA kit and Qubit fluorometer and DNA 12000 kit on the 2100 Bioanalyzer (Agilent). To obtain the longest possible SMRTbell libraries for sequencing (as recommended by the manufacturer), a further size selection step was performed using the PippinHT PFGE system (Sage Science), enriching for the SMRTbell libraries >15 kb for loading onto the instrument. Sequencing primer and P6 polymerase were annealed and bound to the SMRTbell libraries, and each library was sequenced using a single SMRT cell on the PacBio RSII sequencing system with 240 min movies. We combined all subreads from the fastq outputs in Analysis_results from the SMRT Analysis Suite for each isolate.

### Read preparation and hybrid assembly

ONT fast5 read files were base-called with Albacore (v2.0.2, https://github.com/JGI-Bioinformatics/albacore), with barcode demultiplexing and fastq output. Adapter sequences were trimmed with Porechop (v0.2.2, https://github.com/rrwick/Porechop). Read quality was calculated with nanostat (v0.22, https://github.com/wdecoster/nanostat) [21].

Long reads from both ONT and PacBio were prepared using four alternative strategies:

- **Basic**: no filtering or correction of reads (i.e. all long reads available used for assembly).
- **Corrected**: long reads were error-corrected and subsampled (preferentially selecting longest reads) to 30–40× coverage using Canu (v1.5, https://github.com/marbl/canu) [7] with default options.
- **Filtered**: long reads were filtered using Filtlong (v0.1.1, https://github.com/rrwick/Filtlong) by using Illumina reads as an external reference for read quality and either removing 10 % of the worst reads or by retaining 500 Mbp in total, whichever resulted in fewer reads. We also removed reads shorter than 1 kb and used the --trim and --split 250 options.
- **Subsampled**: we randomly subsampled long reads to leave approximately 600 Mbp (corresponding to a long-read coverage around 100×).

Hybrid assembly for each of the two long-read sequencing technologies and for each of the four read processing strategies (for a total of eight hybrid assemblies per isolate) was performed using Unicycler (v0.4.0, https://github.com/rrwick/Unicycler) [13] with default options.

We used Bandage (v0.8.1, https://github.com/rrwick/Bandage) [22] to visualize assemblies, and the Interactive Genome Viewer (IGV, v2.4.3, http://software.broadinstitute.org/software/igv) [23] to visualize discrepancies in assemblies produced by the different methods.

To simulate the effect of additional multiplexing on ONT data and assembly (with current kits allowing for up to 12 isolates to be indexed), we randomly subsampled half or one-third of the ONT reads from each isolate and repeated the assembly as in the ‘Basic’ strategy above. We also subsampled down to a coverage of ~10× for each isolate (based on the genome size from previous assemblies, corresponding to ~5 % of the long reads for each isolate) and repeated the assembly.

Assemblies completed in all cases, apart from two which were both ONT+Illumina hybrids: MGH78578 reference strain (filtered strategy) and RBHSTW-00123 (corrected strategy). Runtimes ranged from 26 to 130 h for Unicycler on the full data with four cores and no downsampling, to approximately 2 h for the ~10× coverage data (range: 1.5–3 h).

### Long-read-only assembly

An alternative method to hybrid assembly is long-read-only assembly, followed by polishing of the genome with short reads to improve sequence quality. We therefore sought to demonstrate that hybrid assembly produced consistent results with this method. While platform-specific software exists, we restricted our choice to open-source platform-agnostic software optimized for plasmid assembly. On the basis of a benchmarking study of long-read assemblers including simulation and five samples from this dataset [24], we used Flye (v2.4.2-release, https://github.com/fenderglass/Flye) [25] followed by polishing using Illumina reads with Pilon (v1.22, https://github.com/broadinstitute/pilon) [26]. We used Flye with 16 cores (-t 16), an estimated genome size of 5 Mb (-g 5 m) (actual range of observed genome sizes: 4.96–6.64 Mb, median of 5.31 Mb) and specific options intended to improve the assembly of plasmids (--plasmids --meta). We used default parameters for Pilon.

### Assembly comparison

We used multiple strategies to compare the features of different hybrid assemblies of the same DNA extract. We assessed all assemblies using CheckM (v1.0.7, https://github.com/Ecogenomics/CheckM) [27] with pplacer and guppy (v1.1.alpha17-6, https://matsen.fhcrc.org/pplacer) [28] using 43 marker genes to assess assembly quality, with the lineage-specific workflow (lineage_wf) applied to each isolate. The results indicated that all assemblies had high completeness (>99 %) and low contamination (median: 0.54%, range: 0.05–2.28 %). The contamination metric is correlated with the total number of estimated circular structures in an assembly (Pearson’s *r*=0.44, *P*=0.053). We believe this may be potentially due to genomic components (e.g. mobile genetic elements) which may exist in the CheckM reference database only in one species but can be present across *Enterobacteriaceae *. CheckM does not directly provide any information on the structural completeness of a genome, and for each isolate all different approaches showed identical CheckM completeness scores despite different numbers of contigs. Therefore, we also considered alternative measures more relevant to structural completeness.

First, we considered the ‘circularity’ of an assembly, i.e. whether contigs in the assembly were identified as circular structures. Circular structures typically represent completely assembled bacterial chromosomes and plasmids; circular structures from different assemblies in our 20 isolates tended to agree in the majority of cases (Table 1) and agreed with the structures of reference genomes for the two reference strains (CFT073 and MH78578). We therefore also used the number of circular contigs in an assembly as a measure of its completeness.

**Table 1.** Summary of all hybrid assemblies in terms of circularized contigs Different rows refer to different isolates. ‘*n* of *m*’ means that *n* contigs were circular in the assembly out of *m* total contigs. When *n* and *m* are identical, it means that the assembly was considered complete, and these cases are shaded in green. ‘Basic’, ‘Corrected’, ‘Filtered’ and ‘Subsampled’ refer to the strategies of long read preparation (see Methods). ‘NA’ refers to cases where the assembly pipeline repeatedly failed. The true number of circular structures was estimated by inspection.

	ONT (MinION)				PacBio (RSII System)				
Isolate	Basic	Corrected	Filtered	Subsampled	Basic	Corrected	Filtered	Subsampled	True circular structures (estimated)
CFT073 (reference)	1 of 1	1 of 1	0 of 9	1 of 1	0 of 9	0 of 9	0 of 9	0 of 9	1
MGH78578 (reference)	6 of 6	4 of 7	na	6 of 6	4 of 7	2 of 22	2 of 22	2 of 22	6
RBHSTW-00029	3 of 9	3 of 9	3 of 9	3 of 9	3 of 9	3 of 9	3 of 9	3 of 9	4
RBHSTW-00053	6 of 6	6 of 6	6 of 6	6 of 6	6 of 6	6 of 6	6 of 6	6 of 6	6
RBHSTW-00059	5 of 5	5 of 5	5 of 5	5 of 5	5 of 5	5 of 5	5 of 5	5 of 5	5
RBHSTW-00122	4 of 4	4 of 4	4 of 4	4 of 4	4 of 4	4 of 4	4 of 4	4 of 4	4
RBHSTW-00123	7 of 7	na	7 of 7	7 of 7	5 of 8	4 of 18	4 of 18	4 of 18	7
RBHSTW-00127	5 of 5	5 of 5	5 of 5	5 of 5	5 of 5	5 of 5	5 of 5	5 of 5	5
RBHSTW-00128	4 of 4	4 of 4	4 of 4	4 of 4	4 of 4	3 of 6	3 of 6	3 of 6	4
RBHSTW-00131	4 of 4	2 of 7	4 of 4	4 of 4	3 of 15	4 of 5	3 of 15	2 of 15	4
RBHSTW-00142	7 of 7	5 of 25	7 of 7	7 of 7	4 of 24	4 of 58	4 of 24	4 of 27	7
RBHSTW-00167	9 of 9	5 of 15	10 of 10	9 of 9	4 of 34	3 of 60	3 of 60	3 of 60	9
RBHSTW-00189	6 of 6	6 of 6	5 of 6	6 of 6	5 of 29	5 of 28	5 of 29	5 of 30	6
RBHSTW-00277	2 of 2	2 of 2	1 of 8	2 of 2	1 of 8	1 of 8	1 of 8	1 of 8	2
RBHSTW-00309	4 of 5	5 of 5	5 of 5	4 of 5	5 of 5	4 of 5	5 of 5	5 of 5	5
RBHSTW-00340	3 of 11	3 of 11	4 of 4	4 of 4	2 of 25	2 of 25	2 of 24	2 of 25	4
RBHSTW-00350	2 of 2	2 of 2	2 of 3	2 of 2	2 of 2	2 of 2	2 of 2	2 of 2	2
RHB10-C07	1 of 1	1 of 1	1 of 1	1 of 1	1 of 1	1 of 1	1 of 17	1 of 1	1
RHB11-C04	3 of 3	3 of 3	3 of 3	3 of 3	3 of 3	3 of 3	3 of 3	3 of 3	3
RHB14-C01	1 of 12	1 of 12	1 of 15	1 of 12	1 of 15	1 of 15	1 of 15	1 of 15	2
Total contigs	109	130	115	102	218	294	276	265	87
Total circularized contigs (% over total estimated circular structures from Bandage: *n*=87 for all isolates)	83 (95 %)	67 (84 %)	77 (95 %)	84 (97 %)	67 (77 %)	62 (71 %)	62 (71 %)	61 (70 %)	
Total circularized contigs for reference strains [i.e. structures known, total *n*=1 (* E. coli *)+6 (* K. pneumoniae *)]	7 (100 %)	5 (71 %)	0 (0 %)	7 (100 %)	5 (71 %)	2 (29 %)	2 (29 %)	2 (29 %)	
Total isolates with all contigs circularised (% isolates)	16 (80 %)	12 (60 %)	13 (65 %)	17 (85 %)	9 (45 %)	7 (35 %)	7 (35 %)	8 (40 %)	

A common error associated with long-read-based assemblies is indel errors, which can artificially shorten proteins by introducing premature stop codons or frameshift errors [29]. To check this possibility we annotated genomes with Prokka (v1.13.3, https://github.com/tseemann/prokka) [30] then aligned all proteins to the full UniProt TrEMBL database (15 November 2018) using DIAMOND (v0.9.22, https://github.com/bbuchfink/diamond) [31] and compared the length of each protein to its top hit. We compared proteins in assemblies for the same sample with Roary (v3.12.0, https://sanger-pathogens.github.io/Roary) [32].

We additionally compared different hybrid assemblies of the same extract using:

- ALE (https://github.com/sc932/ALE) [33], which assesses the quality of different assemblies using a likelihood-based score of how well Illumina reads map to each assembly. ALE was run with default parameters; Illumina reads were mapped to references using Bowtie2 (v2.3.3, https://github.com/BenLangmead/bowtie2) [34].
- DNAdiff (as part of MUMMER v3.23, https://github.com/mummer4/mummer) [35], which compares assemblies of the same strain to detect differences such as SNPs and indels. DNAdiff was run with default parameters on the fasta assembly files.
- REAPR (v1.0.18, https://www.sanger.ac.uk/science/tools/reapr) [36], which (similarly to ALE) evaluates the accuracy of assemblies using information from short read mapping to the assembly. REAPR was run using the options ‘facheck’, ‘smaltmap’ and ‘pipeline’ with default parameters.
- Minimap2 (v2017-09-21, https://github.com/lh3/minimap2) [37] was used to map long reads to the hybrid assemblies, and the mappings were evaluated to compare assembly quality and long read features (identity and length) using scripts from the Filtlong package. We considered the average identity for each base; if there were multiple alignments at a base, we used the one with the best score. We aligned PacBio and ONT reads to the hybrid assemblies obtained either from all PacBio reads or from all ONT reads. Read alignments were classified as: ‘good’ if they had at least one alignment covering 97 % of the read, as a putative ‘chimera’ if they had multiple inconsistent alignments represented by at least 10 % of the read length and ≥70 % nucleotide identity, and ‘other’ if they did not fall into either of the two previous categories.

## Results

### Sequencing data quality

For Illumina data, a median of 2 457 945 [interquartile range (IQR): 2 073 342–2 662 727] paired reads was generated for each isolate, with a median insert size of 363 bp (351–369 bp). The GC content per isolate varied, as expected, by genus (median 53 %, range: 50–57 %), but was consistent with the expected GC content for each isolate based on its species (Table S1, available in the online version of this article).

The PacBio SMRT sequencing data resulted in a median of 160 740 (IQR: 153 196–169 240) sub-reads with median sub-read length of 11 050 bp (IQR: 10 570–11 209 bp) per isolate. Each isolate was sequenced using one SMRT cell on the RSII sequencing system, generating a median of 1.32 Gb (IQR: 1.25–1.36) of data per isolate, with isolates being run in batches of eight (Figure S1, Table S1). For the ONT data, a median of 102 875 reads (IQR: 70 508–143,745 reads) were generated for each isolate, with a median Phred score of 11.8 (IQR: 11.4–12.3). ONT reads had a median length of 14 212 bp (IQR: 13 369–16 267 bp). A median of 13.8 Gb (IQR: 10.8–14.7 Gb) of data was generated per run, resulting in a median of 3.45 Gb per isolate (four isolates multiplexed per run) (Figure S1, Table S1). After hybrid assembly, the mean percentage identity and identity N50 for reads aligned against their respective assemblies were higher for ONT reads than PacBio reads (mean±sd read alignment identity: 86±7 vs. 78±17; Figure S2, Table S2).

### Reference strain assembly comparisons

We compared *de novo* assemblies with published reference genomes for the two reference strains. We also mapped Illumina reads to the published reference genome (see Methods). For CFT073, there were 509 variant sites with respect to the reference genome after mapping Illumina reads. Of these, 211 contained an N in the reference, but 298 sites showed discordancy with the reference genome caused by either: (i) strain evolution in storage and subculture since the reference strain was originally sequenced, (ii) errors in the reference sequence or (iii) errors in mapping. Of these variant sites, 28 (9.4 %) were heterogeneous sites (major allele frequency <0.9), indicating a mixed population in the isolate. Of the remaining 270 discordant sites, 243 (90 %) did not have the (discordant) reference allele in the hybrid assemblies (within one base). For MGH78578, there were 35 variant sites with respect to the reference genome after mapping Illumina reads, and 29 of these (82.8%) were recovered in the hybrid assemblies.

Comparing assemblies to each other using DNAdiff showed that hybrid assembly with Unicycler produced very consistent results across PacBio and ONT (Table S3) and made clear the advantages and disadvantages of each method. For example, while for both strains the ONT Flye assemblies polished with Pilon had a similar number of SNPs relative to the reference as the ONT+Illumina hybrid, they had over ten times as many indels (and, for CFT073, many more unaligned bases). The PacBio Flye assemblies polished with Pilon had the lowest number of SNPs relative to the reference genomes, but they had more unaligned bases than the PacBio+Illumina hybrids for both strains. The most similar assembly to the reference MGH78578 sequence was the PacBio Flye/Pilon assembly (five GSNPs, 36 GIndels), but it had two missing plasmids (8 874 unaligned i.e. absent bases).

### Comparison to long-read-only assembly

We compared hybrid assembly with Unicycler to long-read-only assembly with Flye, followed by polishing using Illumina reads with Pilon (see Methods). For 18 out of 20 isolates the CheckM results for PacBio+Illumina hybrids were identical to those of the respective PacBio-only assemblies followed by Illumina polishing (Table S4). One PacBio Flye assembly had unusually low completeness compared to hybrid assembly (RHB10-C07: 96.88 % vs. 99.93 %) and another isolate had higher completeness (RHB11-C04: 99.89 % vs. 99.62 %). Overall, we observed high consistency between assemblies for each isolate (Figure S3). Noticeably, ONT-only assembly followed by Illumina polishing was inferior and was an outlier compared to the hybrid assemblies for both PacBio and ONT.

### Hybrid assembly comparisons

Using ONT+Illumina hybrid assembly approaches, we were able to completely assemble (i.e. all contigs circularized) the majority of genomes [between 12 (60%) and 17 (85 %)depending on the preparation strategy for long reads, Table 1] without any manual intervention (18 across all strategies). With PacBio+Illumina fewer assemblies were complete [between seven (35 %) and nine (45 %)]. More contigs were also circularized with ONT than with PacBio [up to 84 (97 %) vs. 67 (77 %)], and assemblies were less fragmented (a minimum of 102 total contigs across all 20 isolates for ONT vs. a minimum of 218 for PacBio).

On the basis of the minimap2/Filtlong comparisons (see Methods), most reads from both long-read platforms had ‘good’ alignment to their respective assemblies (~103 000 reads on average for PacBio vs. ~99 000 reads for ONT, Figure S4, Table S5), with slightly more alignments classified as ‘chimeras’ (4 502 vs. 1074 reads) and a much larger number of alignments that were poor and classified as ‘other’ (54 449 vs. 8 222) for PacBio compared to ONT reads (Figure S4, Table S5).

**Fig. 1.**
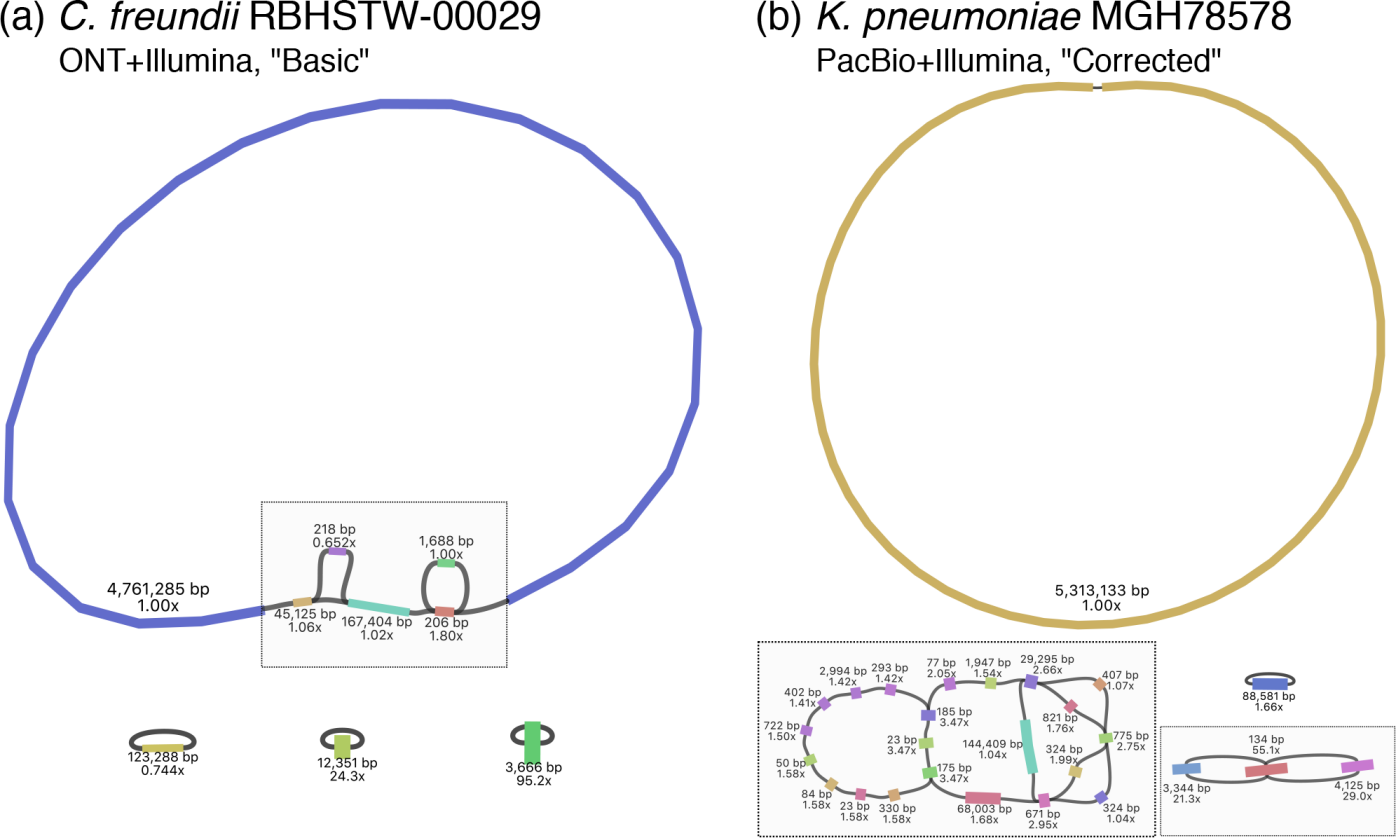
Examples of genome structure uncertainty in hybrid assemblies in (a) the chromosome and (b) the accessory genome. (a) An ONT+Illumina hybrid assembly for isolate RBHSTW-00029 using the ‘Basic’ long-read preparation strategy. (b) A PacBio+Illumina hybrid assembly for isolate MGH78578 using the ‘Corrected’ long-read preparation strategy. Plots were obtained using Bandage on the ‘assembly.gfa’ output file from Unicycler, with grey boxes indicating unresolved structures. Each contig is annotated with contig length and Illumina coverage; connections between contigs represent overlaps between contig ends. The assembly for RHBSTW-00029 in (a) and that of isolate RHB14-C01 (which showed a similar pattern of chromosome structure uncertainty) represented the only two datasets that could not be completely assembled with any of the attempted strategies using ONT+Illumina data. They were also not fully assembled by any PacBio+Illumina strategy, which similarly failed to completely assemble isolates RBHSTW-00189, RBHSTW-00277, RBHSTW-340 and CFT073 (Figure S4). The pattern in (b) was only observed for PacBio+Illumina data, and was the reason for incomplete assemblies for isolates RBHSTW-00123, RBHSTW-00131, RBHSTW-00142, RBHSTW-00167 and MGH78578 (Figure S5).

Some chromosomal regions proved hard to assemble with both PacBio and ONT, e.g. for isolates RBHSTW-00029 and RHB14-C01, but one of the noticeable differences between the two methods was the ability of ONT to resolve repeats on small plasmids (see Fig. 1 and Figure S5). The DNA fragment size selection process used to optimize PacBio sequencing and recommended by the manufacturer may have contributed to this (see Methods), making the assembly of small plasmids reliant on the Illumina short-read component of the dataset only. This is mostly unproblematic due to the typically high coverage of such plasmids, but the presence of repeated structures can make it impossible to resolve them fully using Illumina reads only.

While correcting ONT reads with Canu or filtering them with Filtlong improved assembly completeness for one isolate (RBHSTW-00309), in most cases avoiding this ONT read correction and filtration led to better results (Table 1). This might be due to correction and filtration steps removing reads in a non-uniform way across the genome, and in particular from regions that are already hard to assemble. An alternative strategy deployed to reduce the computational burden of hybrid assembly was to randomly subsample long reads until a certain expected coverage was reached. Table 1 shows that this strategy was preferable to read correction and filtration: it did not reduce assembly completeness but did reduce computation time.

**Fig. 2.**
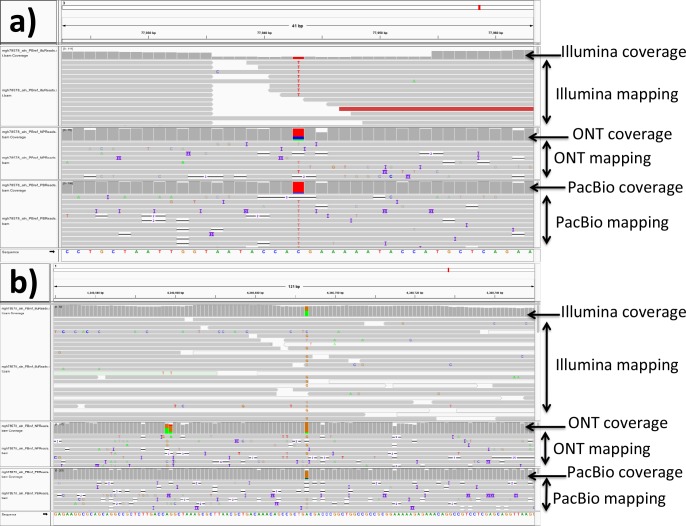
Examples of mismatches identified between the ONT-based and the PacBio-based assemblies for the two reference strains (*E. coli* CFT073 and *K.* *pneumoniae* MGH78578). Each sub-figure is an IGV (v2.4.3) view of part of the PacBio-based assembly, centred around a PacBio-ONT SNP, with all reads from the same isolate mapped to it. We performed this analysis for all SNPs in isolates MGH78578 and CFT073, and report examples for the two most typical patterns observed. (a) SNP from MGH78578 with very low Illumina coverage, but normal PacBio and ONT coverage. Most of the Illumina reads have a different base than the one in the PacBio-assembled reference (the red T's), suggesting perhaps an error in the PacBio assembly. A similar pattern is observed in 14 SNPs in CFT073 (with 12 due to error in the PacBio assembly), and 11 SNPs in MGH78578 (with 10 due to error in the PacBio assembly). (b) SNP from MGH78578 with normal Illumina coverage; Illumina reads support both bases with similar proportions, suggesting that this could be a polymorphic site within the original DNA sample. This pattern was observed for four SNPs in CFT073 and for 13 SNPs in MGH78578.

The analysis of local sequence assembly quality was inconclusive, showing inconsistent results across different methodologies (Table 2), suggesting neither approach was clearly superior to the other in this respect. However, detailed investigation of SNPs between ONT- and PacBio-based assemblies for the reference isolates demonstrated two specific patterns of assembly differences. First, some positions (17 SNPs across the two reference isolates) appeared plausibly polymorphic in the original DNA sample and were called differently in different assembly runs (see Fig. 2a). Second, positions within regions with extremely low Illumina coverage (see Fig. 2b) could have led to assembly errors (25 SNPs across the two reference isolates), the PacBio assemblies being more affected (22 cases vs. three for ONT).

**Table 2.** Comparison between PacBio- and ONT-based hybrid assemblies Comparisons are shown using ALE, DNAdiff and REAPR (see Methods). Different rows represent different isolates. All entries representing a better score for the PacBio assembly are shaded in red, while those showing a better score for ONT are shaded in blue. ‘ALE score’ is the assembly likelihood difference (calculated by ALE from the mapping of Illumina reads) between PacBio and ONT assemblies. ‘Unmapped reads’ refers to the number of Illumina reads that ALE did not map to the corresponding assembly. ‘REAPR errors’ refers to the assembly errors found by REAPR by mapping Illumina reads to the corresponding assembly. For each isolate, one ONT and one PacBio-based assembly with the best completion (i.e. number of circularized contigs) were chosen for comparison. DNAdiff results show the median (range) results from comparing all assemblies for an isolate across read preparation strategies, i.e. 4×4=16 comparisons for each isolate. ‘GSNPs’/‘GIndels’ refer to high-confidence SNPs/indels between ONT and PacBio assemblies

Isolate	ALE score	PacBio unmapped reads (% total)	ONT unmapped reads (% total)	PacBio REAPR errors	ONT REAPR errors	DNAdiff GSNPs	DNAdiff GIndels
CFT073 (reference *E. coli *)	−17 928	29 246 (0.89 %)	29 240 (0.89 %)	5	5	1 (0–1)	0 (0–0)
MGH78578 (reference *K. pneumoniae *)	−1 532 602	41 793 (1.31 %)	38 371 (1.21 %)	8	7	6 (1–7)	0 (0–1)
RBHSTW-00029	207 465	50 056 (1.85 %)	49 876 (1.84 %)	3	3	0 (0–0)	0 (0–0)
RBHSTW-00053	4 727	50 860 (1.62 %)	50 861 (1.62 %)	12	11	1.5 (0–4)	0 (0–0)
RBHSTW-00059	−143 627	37 357 (1.04 %)	36 251 (1.01 %)	15	14	0 (0–0)	0 (0–0)
RBHSTW-00122	0	24 355 (1.18 %)	24 355 (1.18 %)	6	7	0 (0–0)	0 (0–0)
RBHSTW-00123	−1 963 188	56 224 (1.68 %)	57 074 (1.70 %)	17	21	4 (1–6)	4.5 (2–6)
RBHSTW-00127	−1 145	34 206 (0.98 %)	34 206 (0.98 %)	16	16	0 (0–0)	0 (0–0)
RBHSTW-00128	3 114	31 526 (1.06 %)	31 507 (1.05 %)	6	8	2 (1–2)	2 (1–4)
RBHSTW-00131	399 368	25 880 (0.88 %)	26 271 (0.89 %)	24	28	3 (1–7)	1 (1–3)
RBHSTW-00142	−790 773	34 684 (1.23 %)	32 590 (1.16 %)	12	12	3 (1–11)	0 (0–1)
RBHSTW-00167	4 083 063	34 510 (1.13 %)	76 805 (2.52 %)	24	33	21 (18–47)	1.5 (0–4)
RBHSTW-00189	−158 523	37 378 (1.25 %)	37 418 (1.25 %)	9	12	11.5 (7–21)	1 (0–2)
RBHSTW-00277	18 417	33 677 (0.99 %)	33 685 (0.99 %)	16	16	2 (0–2)	0 (0–0)
RBHSTW-00309	−518 811	30 704 (0.88 %)	30 327 (0.87 %)	17	36	2 (0–11)	44.5 (0–86)
RBHSTW-00340	−906 675	30 802 (0.87 %)	29 860 (0.84 %)	11	10	2 (0–4)	0 (0–1)
RBHSTW-00350	21 188	28 907 (0.79 %)	28 907 (0.79 %)	12	13	2 (2–4)	5 (0–8)
RHB10-C07	−23 295	27 779 (0.90 %)	27 777 (0.90 %)	22	21	5 (0–17)	0.5 (0–1)
RHB11-C04	12 774	24 879 (0.86 %)	24 881 (0.86 %)	25	25	2 (0–6)	0 (0–0)
RHB14-C01	172 712	30 478 (0.95 %)	30 576 (0.95 %)	13	12	3 (0–3)	0 (0–0)

The proportion of proteins with a length of <90 % of their top UniProt hit was low (∼2–4 %, cf. 3.7 % for the RefSeq assembly of *E. coli* MG1655) and extremely consistent across ONT+Illumina and PacBio+Illumina assemblies (Figure S6), suggesting that indels were not a significant problem in the assemblies. There was very close agreement between methods (median discrepancy <5 proteins), although there were a greater number of cases where more proteins were found in the ONT+Illumina assemblies (Figure S7). Proteins found uniquely in an assembly tended to be found on a contig that was fragmented in the comparison assembly (e.g. the third plasmid in the ONT-based assembly for RBHSTW-00167 was fragmented in the comparison PacBio-based assembly, and was the location of 11 proteins unique to the ONT-based assembly), highlighting that the degree of contig fragmentation in an assembly can affect conclusions about gene presence beyond just the inability to resolve genomic structures (Table S6, Figure S5).

### Effect of long-read coverage on assemblies

We multiplexed four isolates per ONT flow-cell, but further multiplexing is possible, although it can lead to reduced coverage. We therefore investigated the effect of downsampling the numbers of ONT long reads (see Methods). Halving the available reads (equivalent to multiplexing with eight barcodes) had no detectable negative effect on the assemblies (Table S7). Using a third (equivalent to multiplexing with 12 barcodes) slightly increased the fragmentation of the assemblies overall (one fewer completed assembly and nine additional non-circular contigs). However, these results were not uniform: two assemblies had chromosomes that could be circularized only with downsampling (RBHSTW-00309 and RBHSTW-00340). Subsampling to a coverage of ∼10× (see Methods) increased the fragmentation of the assemblies overall, but 65 circular contigs were still circularized and DNAdiff comparisons showed that the assemblies were highly similar to the assemblies from the full data (Figure S3).

### DNA preparation and sequencing costs

Beyond considerations of assembly accuracy, an important and realistic consideration when choosing a sequencing approach is cost. While we do not attempt to calculate estimates that will apply across different labs and settings, we report here our consumables costs per isolate (i.e. exclusive of other potential costs such as laboratory and computational staffing, purchase and maintenance of laboratory and computational infrastructure, service contracts, etc.) in case it is helpful for informing others.

The cost of bacterial culture and DNA extraction was approximately £12 per isolate, resulting in sufficient DNA for all three sequencing methods to be performed in parallel on a single extract. The cost for library preparation and one lane of Illumina HiSeq sequencing containing 192 samples was £7 667 per lane (~£40 per isolate in this study). ONT sequencing was performed by multiplexing four isolates per run, with the library preparation, barcoding and flow cells costing ~£100 per isolate due to lower cost flow cells after a linked purchase of a GridION (excluding this, the cost ranges from £125 to £220 per isolate depending on the flow cell bundle purchased). PacBio sequencing was done using one isolate per library per SMRTcell on the RSII system, with costs of ~£280 per isolate. For all technologies, more recent sequencing instruments and further multiplexing could result in significantly higher throughput, correspondingly reducing the cost per isolate in each case. We cannot precisely estimate these costs here.

To summarize, based on the sequencing we performed, the minimum cost per isolate using the PacBio RSII system to generate a PacBio+Illumina hybrid assembly (~£320) is higher than for generating an ONT+Illumina hybrid assembly (~£165–260). We stress that these costs do not include infrastructural and staffing costs, and that changing factors since the time of these experiments (late 2017) could result in further differences in costs across the two long-read platforms.

## Discussion

Combining short-read Illumina sequencing with different long-read sequencing technologies and using Unicycler, a publicly available and widely used hybrid assembly tool, we found that ONT+Illumina hybrid assembly generally facilitates the complete assembly of complex bacterial genomes without additional manual steps. Our data thus support ONT+Illumina sequencing as a non-inferior bacterial genome hybrid assembly approach compared with PacBio+Illumina, leading to more complete assemblies, and to significantly lower costs per isolate if multiplexed.

We also investigated the impact of different long-read processing strategies on assembly quality and found that different strategies can result in more complete assemblies. We showed that quality-based filtration and correction of long reads can apparently paradoxically result in worse performance than just using unfiltered and uncorrected reads. There is no obvious explanation for this; we speculate that preferential removal of long reads from hard-to-sequence regions might be a contributing factor, but we have been unable to establish if this is the case. We propose a different strategy to reduce the computational burden of hybrid assembly without affecting the final outcome: randomly subsampling long reads down to a desired level of coverage. We demonstrated that this strategy generally results in better assemblies for ONT sequencing data.

PacBio+Illumina hybrid assembly has the advantage that it recovers small plasmids (<10 kb) missed by PacBio-only assembly followed by Illumina polishing. This is almost certainly due to the standard size selection step (as recommended by PacBio, see Methods) which shears reads to a mean length of 15 kb, then enriches for reads >15 kb. With only PacBio reads, there may therefore be a trade-off for complex bacterial genomes: between initially shearing to a longer mean length to improve circularization and altering the size selection step to recover small plasmids.

Although we did not investigate them in detail, we identified some recurrent patterns of local hybrid misassembly that could be systematically addressed in the future. One of these is the presence of polymorphisms in the DNA extract. Sometimes these may represent genuine minor variants present in the isolate, but the salient fact here is that current bacterial assembly methods assume that no position is polymorphic. This can lead to an imperfect representation of the genomic content if this is not the case. We advocate for the inclusion or awareness of polymorphisms within assembly polishing methods (e.g. Pilon [26]). The other problem we identified is that regions with very low Illumina coverage tend to be enriched with small assembly errors. This problem could similarly be addressed in the future with hybrid assembly polishing methods, which would supplement Illumina-based polishing with long-read-based polishing in regions with low Illumina coverage.

There were several limitations to our study. First, we included only two reference strains, and our analyses suggested that the ‘true’ sequences for these had slightly diverged from the publicly available reference sequences. Mapping Illumina reads back to the original reference allowed us to identify those positions that were probably true biological variation after years of storage and/or subculture – a known possibility that has been previously observed for bacterial reference strains (e.g. in archived cultures of *Salmonella enterica* serovar Typhimurium LT2 [38]). However, we still found small discrepancies between the published reference genome and our assemblies, which could be due to either errors in the original reference sequences (first published in 2002 for CFT073, 2007 for MGH78578), or possible errors in our hybrid assemblies. Thus, making comparisons for any given approach is difficult, even in the case where a reference is available. Of note, we tried to minimize biological variability introduced in culture by sequencing the same DNA extract across different platforms. For 18 isolates the ‘true’ underlying sequence was unknown, which is common for highly plastic *Enterobacteriaceae* genomes. There is no consensus on how best to evaluate assemblies and assembly quality when a reference is not available. We therefore used several approaches, and these were not always consistent with each other.

Assemblies can sometimes be further improved after an initial evaluation using manual completion. We did not investigate manual completion for our hybrid assemblies because in our experience it is hard to replicate, has not been benchmarked and validated, is more easily biased, and is not feasible for processing large numbers of isolates (hundreds or thousands). However, it may be appropriate for other research settings. While we compared a hybrid approach to long-read-only assembly with Flye and subsequent polishing with Pilon, which is significantly faster than Unicycler, we did not investigate all possible options for assembly involving long reads, which may represent other potential options for closing assemblies. We did not identify any published, publicly available tools developed to specifically handle PacBio+Illumina hybrid assembly, although some research groups may have implemented and validated these in-house. Finally, we did not investigate the effect of different basecallers. The evolution of both technologies and post-sequencing processing of data generated by both ONT and PacBio platforms is rapid, and recent advances have been made (e.g. in basecalling with the switch from Albacore to Guppy for ONT data). Our assumption is that such advances which improve read quality and basecalling will improve assembly quality, but we have not carried out specific comparisons.

In conclusion, reference-grade, complete hybrid assemblies can be effectively generated for complex bacterial genomes including multiple plasmids. Although hybrid assembly with Unicycler has disadvantages (such as longer runtimes), it gave generally similar results to long-read-only assembly followed by short-read polishing for both ONT and PacBio. It also offers some specific advantages: improved quality (for ONT) and recovery of small plasmids (for PacBio). We have shown that using multiplexing on ONT platforms in combination with Illumina data is a viable option for the routine, automated generation of high-quality reference-grade assemblies. Given the average yields that can be generated with these devices, it is now feasible to comfortably multiplex at least eight *Enterobacteriaceae* isolates per ONT flowcell. At current listed cost prices, this would represent a cost of the order of ~£100 per hybrid assembly (all laboratory and sequencing consumables costs for both Illumina and ONT).

## Data bibliography

1. Raw sequencing data: NCBI BioProject Accession PRJNA422511 (https://www.ncbi.nlm.nih.gov/bioproject/PRJNA422511).

2. Assemblies: FigShare doi https://doi.org/10.6084/m9.figshare.7649051 (https://doi.org/10.6084/m9.figshare.7649051).

3. NCBI GenBank reference sequences:

a. CFT073: NC_004431.1 (chromosome)

b. MGH78578: NC_009648.1 (chromosome); NC_009649.1, NC_009650.1, NC_009651.1, NC_009652.1, NC_009653.1 (plasmids)

## Supplementary Data

Supplementary File 1

Supplementary File 2
