# Supplementary material for: Comparison of long-read sequencing technologies in the hybrid assembly of complex bacterial genomes
Source: Microb Genom. 2019 Sep 4;5(9):e000294. doi: 10.1099/mgen.0.000294 (PMC6807382; doi:10.1099/mgen.0.000294)
Supplement: Supplementary File 1 [file mgen-5-294-s001.pdf]

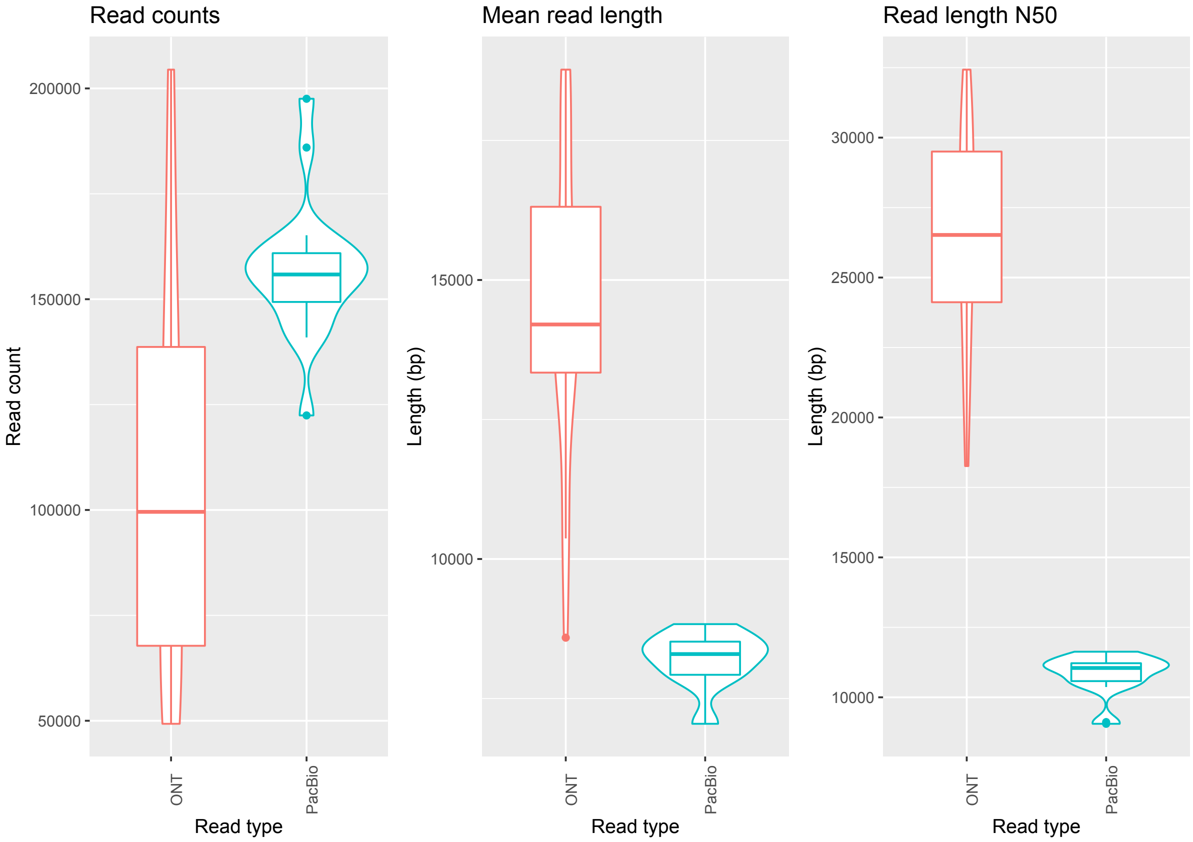

Figure S1. Read counts and read length distributions for ONT and PacBio outputs.

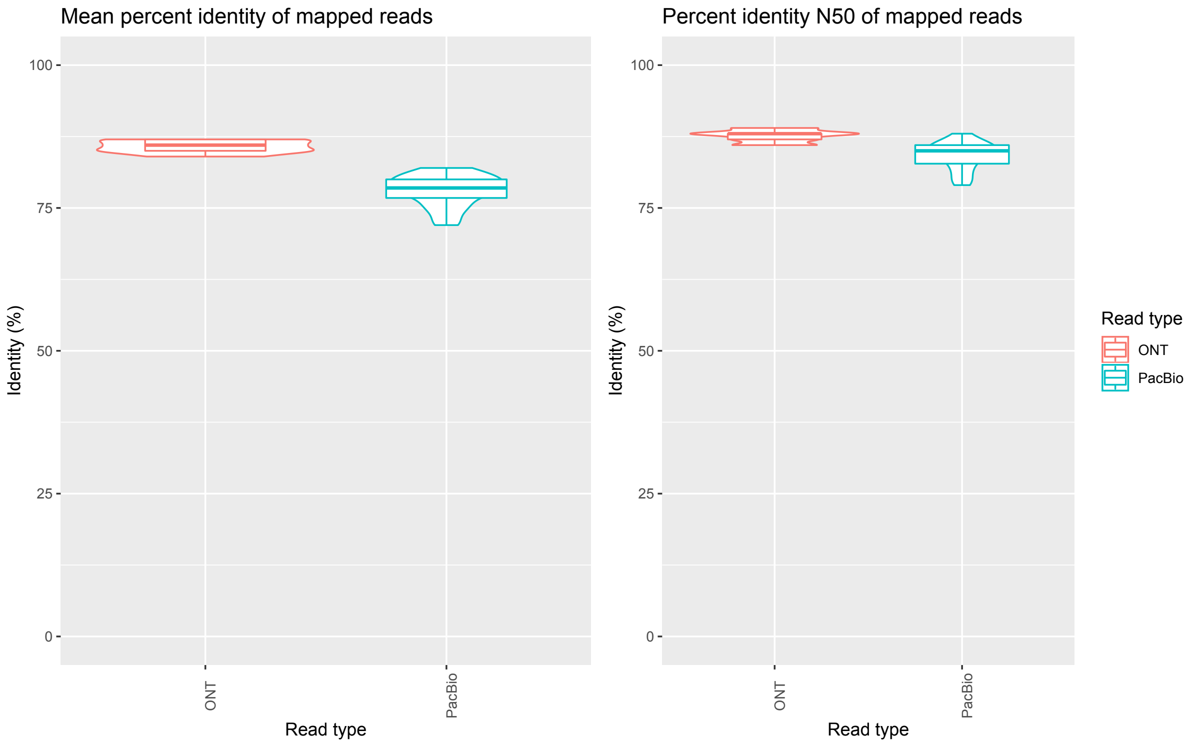

Figure S2. Mean percent identities and identity N50 values of ONT/PacBio reads aligned to the hybrid assemblies. We considered the average identity for each base, and if there were multiple alignments at a base, we used the one with the best score. We aligned PacBio reads to the hybrid assembly obtained from all PacBio reads. We aligned ONT reads to the hybrid assembly obtained from all ONT reads. Identity N50 represents the percent identity for which half of the total bases are in reads with this identity value or higher. Complete statistics are in Table S2.

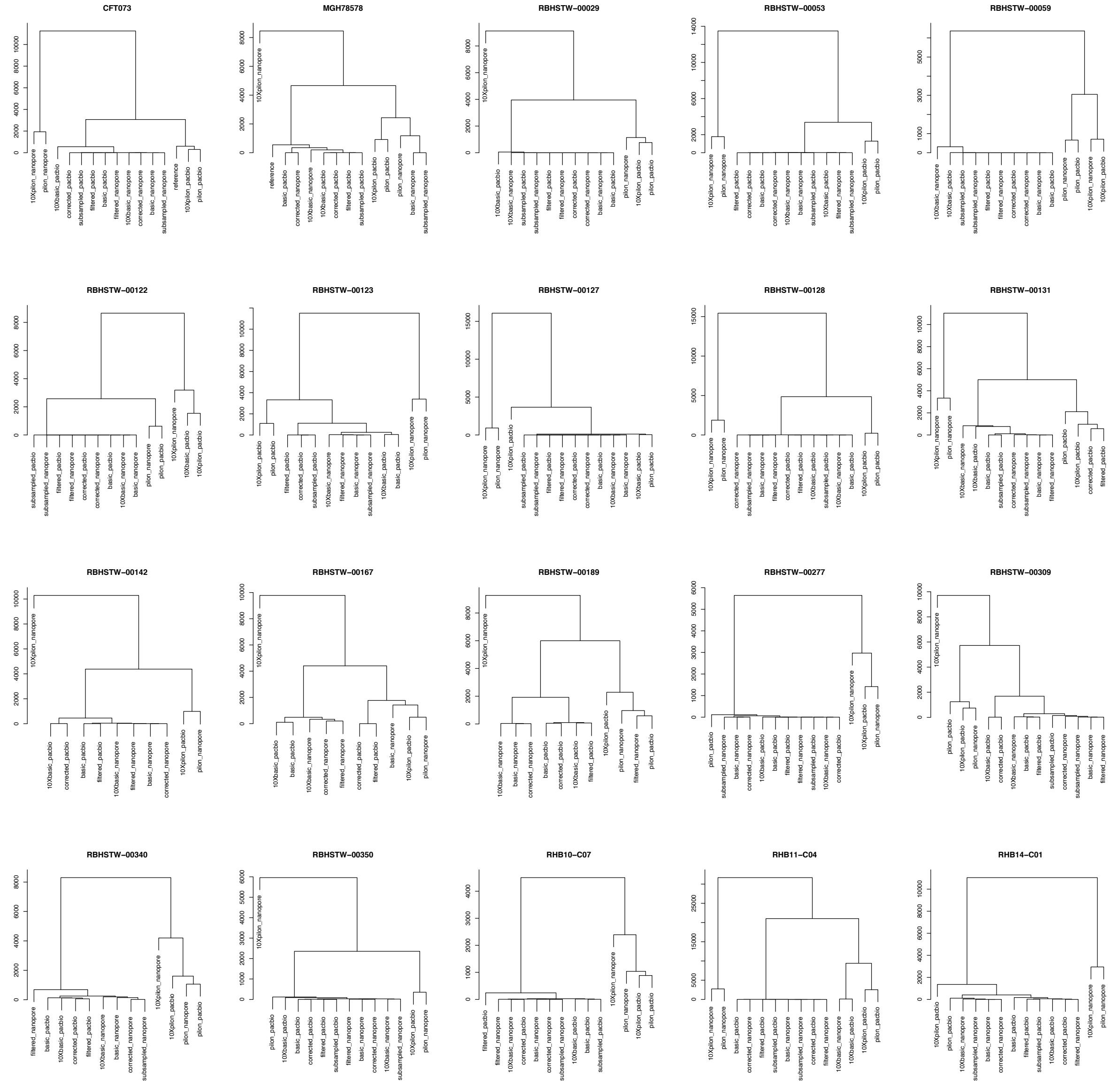

Figure S3. Similarity of assemblies from different approaches. Dendrograms produced using pairwise distances from DNAdiff comparisons of assemblies. For the distance, we used an approximate measure of local assembly sequence similarity: the sum of GSNPs, GIndels, and unaligned bases (reference and query). A '10X' prefix indicates that an assembly is based on long-read data after sampling to approximately 10X coverage (based on the known genome size from the full data assemblies).

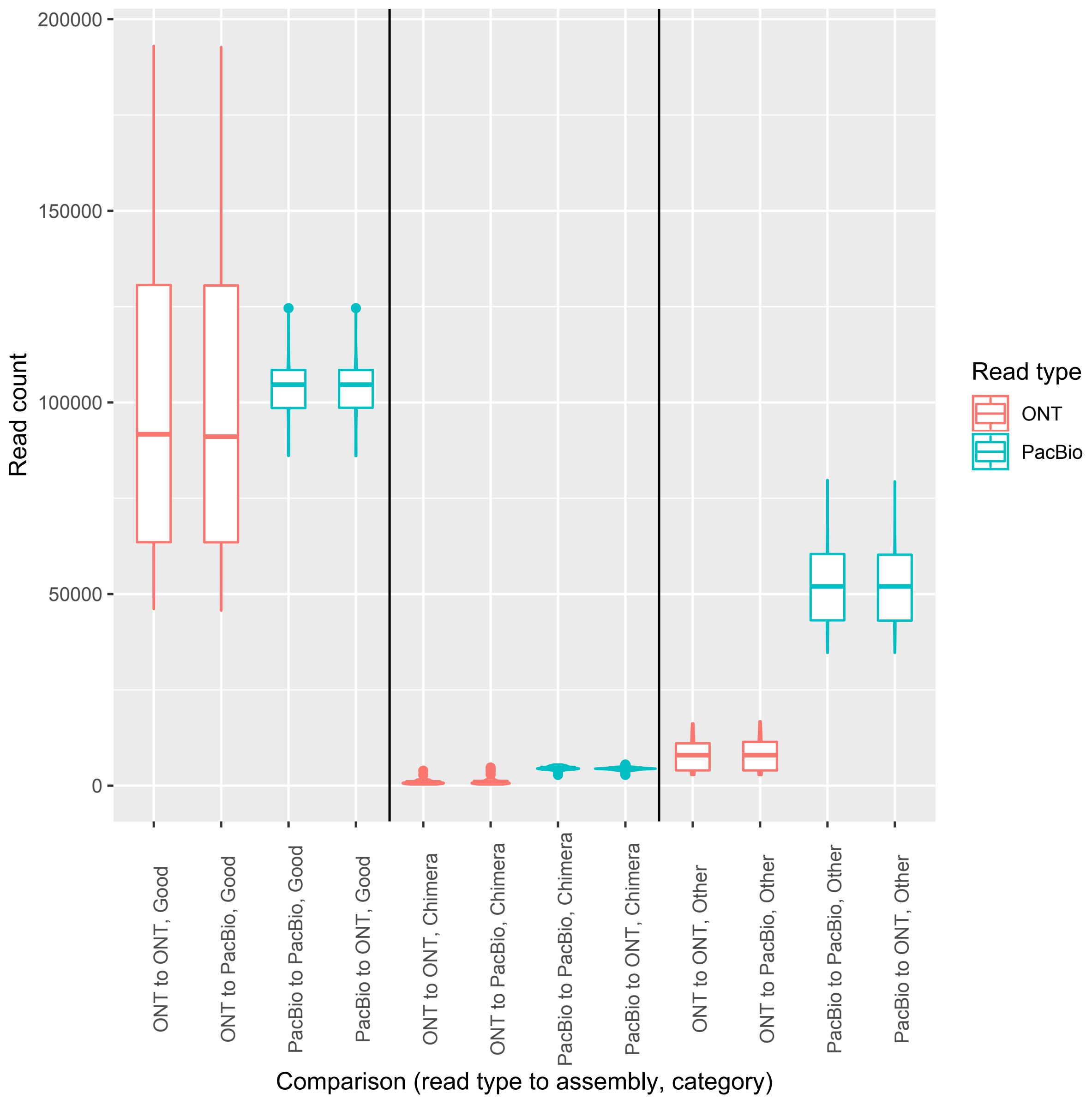

Figure S4. Summary of read-to-assembly alignments. All assemblies considered were obtained using all reads of the given type. Reads are classified as "good" if they have at least one mapping covering 97% of the read. They are classified as a putative "chimera" if they have multiple inconsistent alignments with at least 10% of read length and 70% identity. Complete statistics from minimap2/Filtlong outputs are in Table S5.

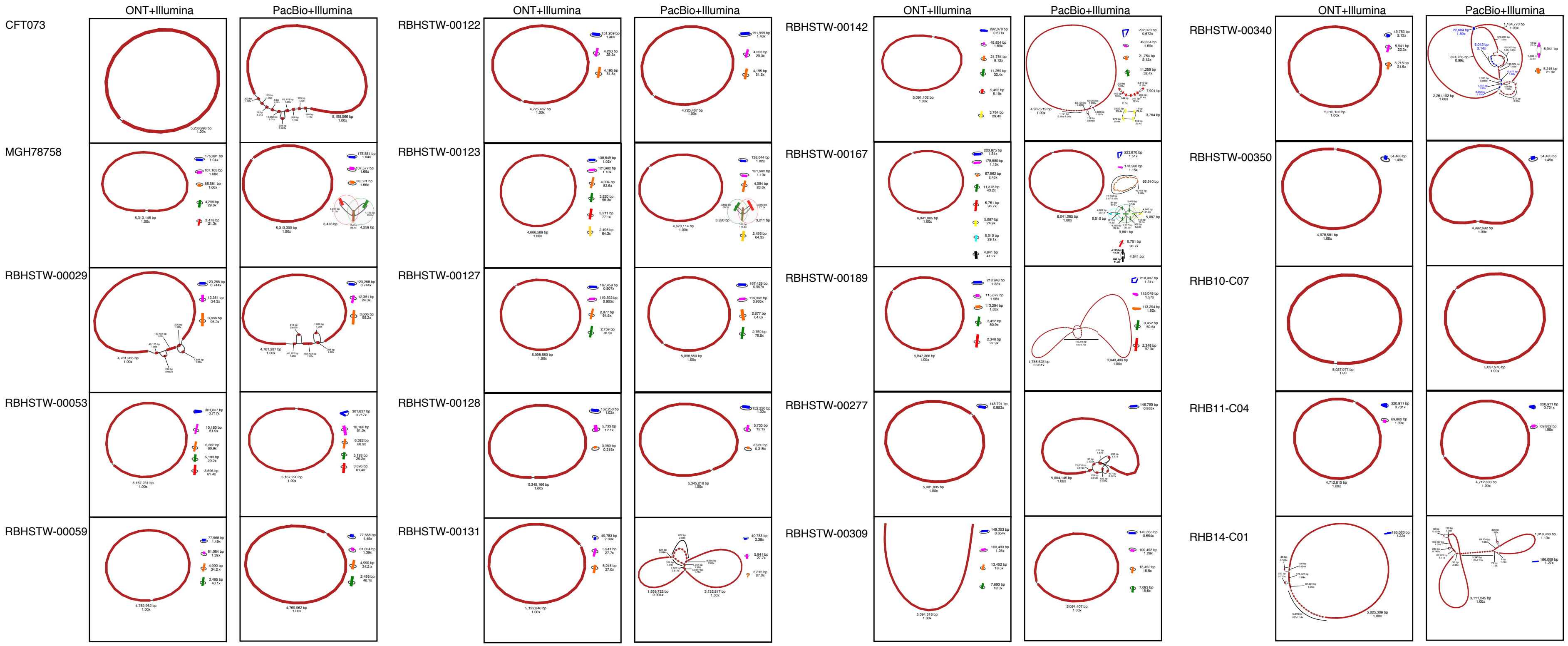

Figure S5. Bandage plots for hybrid assemblies. Each square represents one genome assembly. Shown are the ONT+Illumina (left) and PacBio+Illumina (right) assemblies for each isolate (4 columns of 5 isolates). All assembly plots are for the globally optimal long-read preparation strategy for each sequencing approach i.e. "Subsampled" for ONT+Illumina and "Basic" for PacBio+Illumina (see Methods). Sequential colours for plasmids are for identical structures within isolates, but not between.

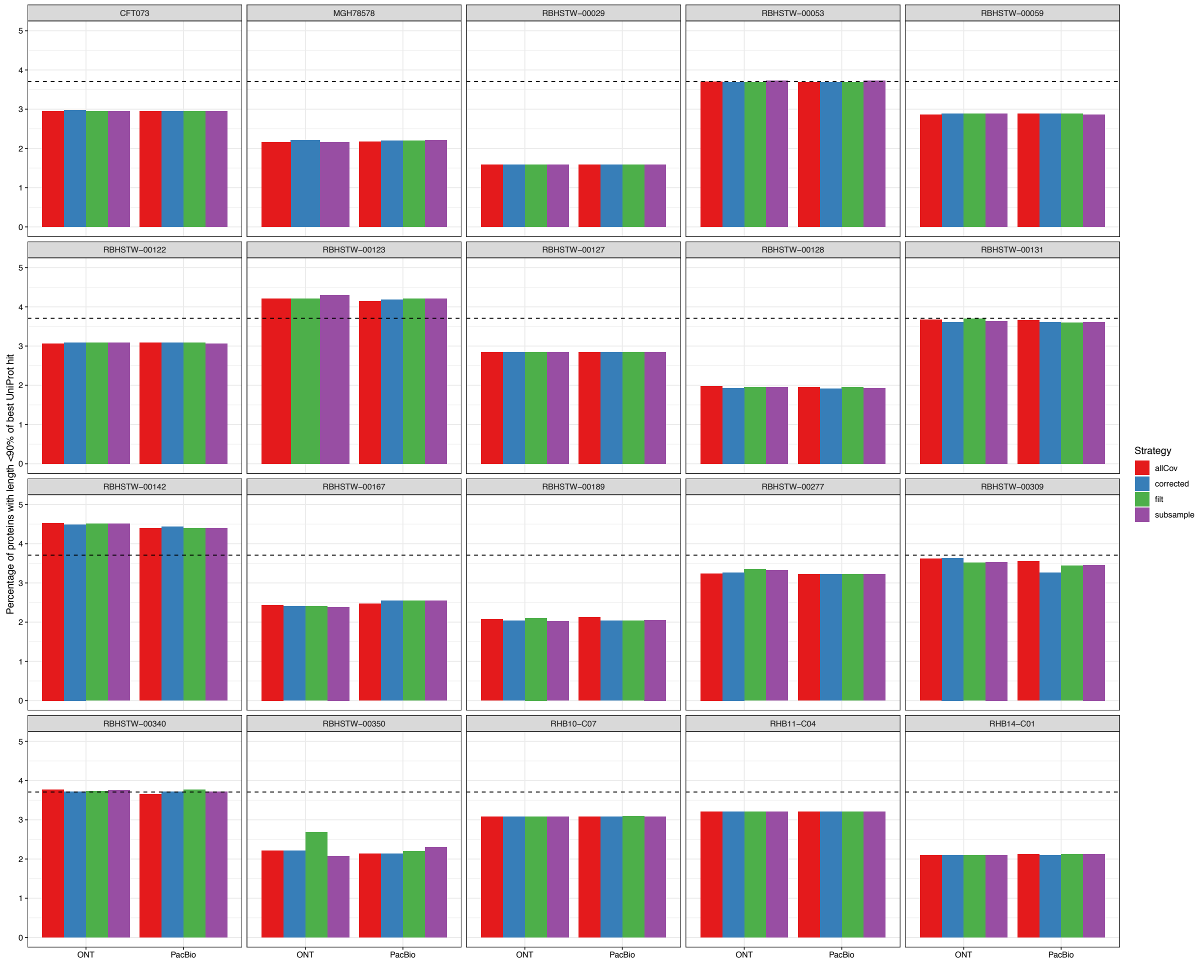

Figure S6. Percentage of proteins with a length <90% of top UniProt hit. Proteins in assemblies were annotated with Prokka then blasted with DIAMOND against the full UniProt database (see Methods). The proportion of proteins with a length <90% of their top UniProt hit gives a simple test for artificially shortened proteins due to indel errors in assembly. The black dashed line indicates the percentage in an existing high-quality reference genome for *E. coli* MG1655 (157 proteins out of 4240; RefSeq GCF_000005845.2). Absolute numbers were all <250; shown here is the value as a percentage of the maximum number of proteins observed in any assembly for the sample to allow comparison between different genome sizes.

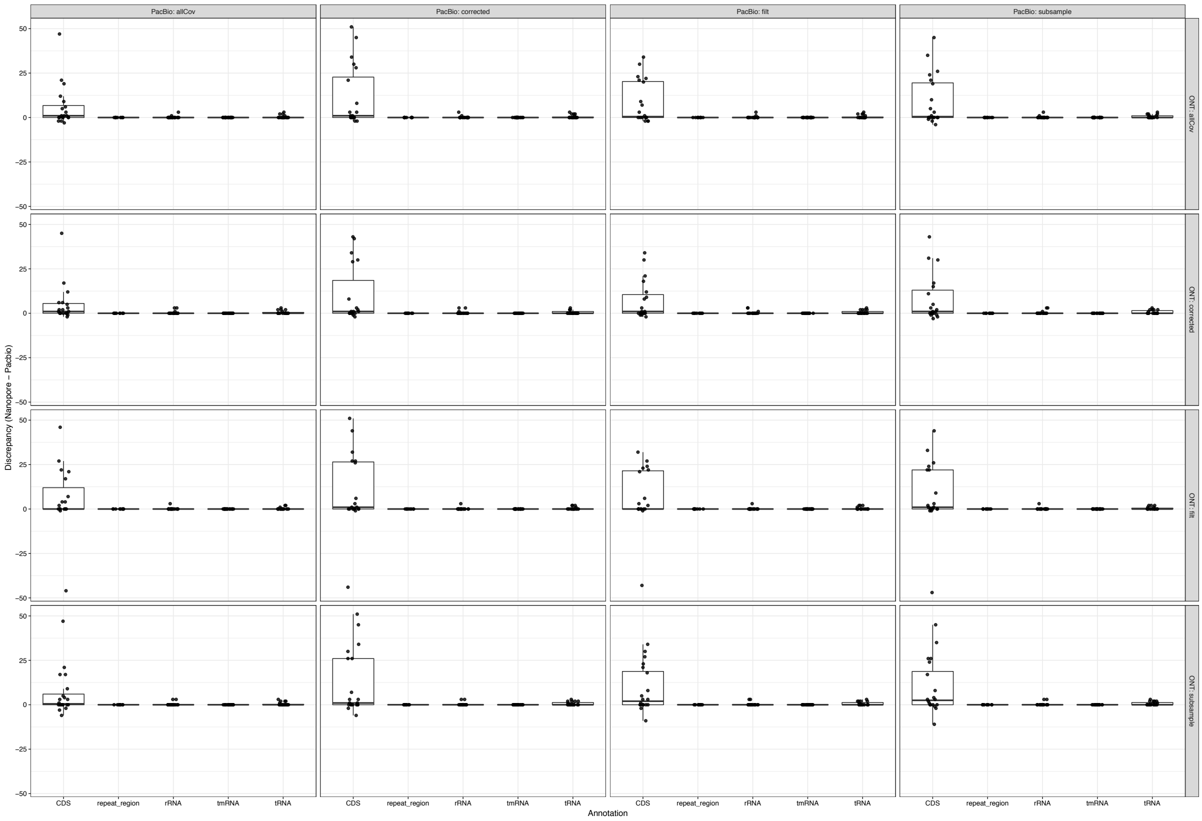

Figure S7. Comparison of discrepancy in total Prokka annotated regions across all assemblies. The discrepancy is the number of annotated regions in the ONT+Illumina assembly minus the number of annotated regions in the PacBio+Illumina assembly. All 4x4=16 comparisons of different long-read preparation strategies are shown.
